# Survival of patients with nonseminomatous germ cell cancer: a review of the IGCC classification by Cox regression and recursive partitioning

**DOI:** 10.1038/sj.bjc.6601665

**Published:** 2004-02-24

**Authors:** M R van Dijk, E W Steyerberg, S P Stenning, E Dusseldorp, J D F Habbema

**Affiliations:** 1 Department of Public Health, Erasmus MC – University Medical Center Rotterdam, PO Box 1738, 3000 DR Rotterdam, The Netherlands; 2 Medical Research Council, Clinical Trials Unit, 222 Euston Road, London, NW1 2DA, UK; 3 Data Theory Group, Department of Education, Leiden University, PO Box 9555, 2300 RB Leiden, The Netherlands

**Keywords:** Cox regression, recursive partitioning, germ cell cancer, prognostic classifications

## Abstract

The International Germ Cell Consensus (IGCC) classification identifies good, intermediate and poor prognosis groups among patients with metastatic nonseminomatous germ cell tumours (NSGCT). It uses the risk factors primary site, presence of nonpulmonary visceral metastases and tumour markers alpha-fetoprotein (AFP), human chorionic gonadotrophin (HCG) and lactic dehydrogenase (LDH). The IGCC classification is easy to use and remember, but lacks flexibility. We aimed to examine the extent of any loss in discrimination within the IGCC classification in comparison with alternative modelling by formal weighing of the risk factors. We analysed survival of 3048 NSGCT patients with Cox regression and recursive partitioning for alternative classifications. Good, intermediate and poor prognosis groups were based on predicted 5-year survival. Classifications were further refined by subgrouping within the poor prognosis group. Performance was measured primarily by a bootstrap corrected *c*-statistic to indicate discriminative ability for future patients. The weights of the risk factors in the alternative classifications differed slightly from the implicit weights in the IGCC classification. Discriminative ability, however, did not increase clearly (IGCC classification, *c*=0.732; Cox classification, *c*=0.730; Recursive partitioning classification, *c*=0.709). Three subgroups could be identified within the poor prognosis groups, resulting in classifications with five prognostic groups and slightly better discriminative ability (*c*=0.740). In conclusion, the IGCC classification in three prognostic groups is largely supported by Cox regression and recursive partitioning. Cox regression was the most promising tool to define a more refined classification.

Testicular germ cell tumours (seminomatous and nonseminomatous) are the most common cancers among young adult men. Since the 1970s, long-term cure rates of patients with germ cell tumours have increased to over 80%, because of the ability of cisplatin-based chemotherapy to cure advanced disease (Bosl and Motzer, 1997; Hartmann *et al*, 1999; Steele *et al*, 1999; Sonneveld *et al*, 2001). Owing to the high overall cure rate, interest has shifted from increasing the overall cure rate to reducing treatment-related toxicity for patients with a good prognosis (de Wit *et al*, 2001). On the other hand, high-risk patients, eligible for more intensive treatment, for example, stem-cell support or high-dose chemotherapy, should be identified (Bokemeyer *et al*, 1999, 2002).

Several classifications have been proposed in the past to distinguish patients according to prognosis, by identifying and combining the main prognostic factors for progression-free survival (PFS) and overall survival (Bajorin *et al*, 1988, 1991; Mead *et al*, 1992). The coexistence of classifications differing in type, complexity and ability to separate good from poor prognosis complicated international collaboration in randomised trials and made comparison of nonrandomised studies impossible. International collaboration by the International Germ Cell Cancer Collaborative Group resulted in the development of the International Germ Cell Consensus Classification (IGCC classification), which is widely applied and easy to use and remember (IGCCCG, 1997).

For the IGCC classification, readily available risk factors were selected from a wider set following Cox regression analyses, namely primary site, presence of nonpulmonary visceral metastases (NPVM) and elevation of the tumour markers alpha-fetoprotein (AFP), human chorionic gonadotrophin (HCG) and lactic dehydrogenase (LDH). All variables were categorical, since no major differences in performance were found compared to using continuous variables (McCaffrey *et al*, 1998). In Table 1

**Table 1:** International Germ Cell Consensus Classification for nonseminoma

*Good prognosis*
Testis/retroperitoneal primary site=0
and
No nonpulmonary visceral metastases=0
and
AFP good=0 and HCG good=0 and LDH good=0
**Max=0**

*Intermediate prognosis*
Testis/retroperitoneal primary site=0
and
No nonpulmonary visceral metastases=0
and
AFP intermediate=1 or HCG intermediate=1 or LDH intermediate=1
**Max=1**

*Poor prognosis*
Mediastinal primary site=2
or
Nonpulmonary visceral metastases=2
or
AFP poor=2 or HCG poor=2 or LDH poor=2
**Max=2**

Tumour markers AFP/HCG/LDH: Good – AFP <1000 ng ml^−1^, HCG <5000 iu l^−1^, LDH <1.5 × upper limit of normal; Intermediate – AFP 1000–10000 ng ml^−1^, HCG 5000–50000 ng ml^−1^, LDH 1.5–10 × N; Poor – AFP >10000 ng ml^−1^, HCG >50000 iu l^−1^, LDH >10 × N.

, how the risk factors were combined into three prognostic groups for patients with nonseminomatous germ cell tumours (NSGCT) with either good, intermediate or poor prognosis are shown. The good prognosis group is characterised by the absence of adverse risk factors. The intermediate prognosis group is defined by the presence of any intermediate tumour marker, that is, one or more intermediate tumour markers are present. The poor prognosis group is characterised by the presence of any of the poor risk factors mediastinal primary site, NPVM, AFP poor, HCG poor or LDH poor, that is, one or more poor risk factors are present. The classification can be seen as a max function where the good, intermediate and poor prognosis groups have a maximum score of zero, one or two, respectively.

In the IGCC classification, all intermediate tumour markers and all poor risk factors were required only to be sufficiently bad to be classified as intermediate and poor prognosis, respectively, that is, differences in importance between intermediate tumour markers and differences in importance between poor risk factors are not taken into account. Furthermore, no distinction is made between the number of intermediate tumour markers in the intermediate prognosis group and the number of poor risk factors in the poor prognosis group. Better discrimination might be achieved by incorporating differences in predictive strength and testing specific interaction terms.

Furthermore, it is difficult to adjust the current classification for changes in treatment strategy. A more flexible scoring system could more easily identify subgroups for the identification of very high risk patients eligible for novel chemotherapy approaches such as high-dose chemotherapy or the use of novel cytotoxic agents (Bokemeyer *et al*, 1999; Kollmannsberger *et al*, 2000). We however note that an important consideration in developing the IGCC classification was that all the prognostic groups should be large enough to make randomised trials of new treatments for each prognostic group feasible (IGCCCG, 1997).

The aim of this study was to reconsider steps taken in the development of the IGCC classification, and to investigate alternative classifications based on Cox regression and recursive partitioning (Breiman *et al*, 1984) that may discriminate better and be more suitable to identify more subgroups.

## MATERIALS AND METHODS

### Patients

Centres participating in the International Germ Cell Collaborative Group provided retrospective data of 5202 adult male patients with NSGCT. All patients were treated between 1975 and 1990 with cisplatin-based chemotherapy. Data were collected on age, primary site, date of diagnosis, levels of serum AFP, HCG and LDH, nodal disease in the abdomen, mediastinum, and neck, lung metastases, spread to other visceral sites like liver, bone and brain and on treatment details like previous therapy. For the development of the IGCC classification, patients without missing data on the risk factors primary site, NPVM, tumour markers AFP, HCG and LDH and the outcome survival were selected (*n*=3048) (IGCCCG, 1997).

### Outcome and IGCC risk factors

The outcome measures were PFS and overall survival from the start of the chemotherapy. The risk factors in the IGCC classification were primary site (testis/retroperitoneal *vs* mediastinum), presence of NPVM (yes/no) and tumour markers AFP, HCG and LDH. Each tumour marker had three categories; good, intermediate and poor with specific cutoff points on the continuous tumour markers (see Table 1) (IGCCCG, 1997). The same risk factors and categories were used to construct the alternative classifications based on Cox regression and recursive partitioning.

### Statistical analyses

The IGCC classification makes no clear distinction between the intermediate tumour markers and between the poor risk factors and is represented by a max score. One way to assess this assumption is by evaluating whether the weights in the IGCC classification were optimally allocated to the risk factors. We hereto varied the IGCC weights (1/2) over the levels of the risk factors and compared all possible combinations with respect to performance. Performance was quantified by the difference in minus twice the log likelihood (model *χ*^2^) (Clayton and Hills, 1993).

We used the Cox regression to study the univariable and multivariable effects of the IGCC risk factors on the overall survival, expressed as Hazard ratios and regression coefficients.

The Cox regression model formed the basis of classification ‘5R’. We multiplied the multivariate regression coefficients by 10 and rounded them to obtain weights. A sum score was calculated by multiplying the weights with individual patient characteristics and adding the resulting individual scores (Assmann *et al*, 2002). We calculated the estimated 5-year survival rate for each score.

The IGCC classification can be viewed as implying that the risk factors are strongly dependent, that is, that there are interactions between risk factors. There is, for example, no distinction made between patients with one poor risk factor or three poor risk factors. To test whether and which interactions were present, we added all two-way interactions between the IGCC risk factors in a Cox regression model. Important interactions were selected through stepwise backward selection (*P*<0.05). Since interactions based on small number of patients give unreliable regression coefficients, the interaction terms were defined as linear. The resulting model forms the basis of classification ‘5Ri’. A sum score based on a regression model with interactions is, however, more difficult to calculate and interpret. Therefore, a table was constructed with 5-year survival estimates for all possible combinations of the IGCC risk factors based on the Cox regression model with linear interactions. The number of patients on which each survival estimate was based is given to indicate the reliability of the survival estimates.

An alternative and visually more attractive way of exploring and presenting interactions between risk factors is by growing a tree through recursive partitioning (Breiman *et al*, 1984; Segal and Bloch, 1989; Ahn and Loh, 1994) that we used to construct classification ‘5T’. A binary tree is built by the following process: first the risk factor that best splits the data into two groups, leading to the largest decrease in prediction error, is determined (recursive partitioning or splitting method). Splitting continues until the subgroups reach a minimum size or until no improvement can be made (stopping rule). The full tree, which is often too complex and overfit, is pruned using crossvalidation. All trees within one standard error of the lowest crossvalidated prediction error are considered as equivalent. From these equivalent trees, the simplest is chosen as final tree (Breiman *et al*, 1984).

As a splitting method, the exponential scaling method was used (Therneau *et al*, 1990; LeBlanc and Crowley, 1992). The splitting process stopped when a minimum of five patients per groups was reached or when there was no further decrease in prediction error. We used 10-fold crossvalidation to determine the optimal tree size. Modelling was performed with S-plus version 2000 using the RPART library that contains a recursive partitioning method for survival data.

The RPART library (rpart2.zip) and manual (rpart2doc.zip) can be found at http://www.stats.ox.ac.uk/pub/SWin.

### Prognostic groups

In all classifications, three prognostic groups were identified using the estimated 5-year survival by sum score (classification 5R), combination of risk factors (5Ri) or binary tree (5T). Subgroups with a 5-year survival higher than 90% were considered as good prognosis, between 65 and 89% as intermediate prognosis, and lower than 65% as poor prognosis.

Furthermore for each classification, we explored the possibility of identifying more subgroups. For the IGCC classification, this was carried out by allowing weights to vary from zero to four (instead of zero to two), and comparing all possible combinations on performance. For classifications 5R, 5Ri and 5T, we changed the cutoff points on estimated 5-year survival. A 5-year survival rate higher than 90% was considered as good prognosis, 75–89% as intermediate prognosis, 60–74% as good-poor prognosis, 40–59% as intermediate-poor prognosis, and lower than 40% as poor-poor prognosis (Kollmannsberger *et al*, 2000). Survival of the five groups of the IGCC classification and classifications 5R, 5Ri and 5T was presented by Kaplan–Meier curves.

### Performance

The classifications were evaluated by their ability to distinguish between patients differing in survival. An indication of the discriminative ability is the difference in 5-year survival rates between the good, intermediate and poor prognosis groups. A *c*-statistic was also calculated for both the three and five group classifications. For binary outcomes, the *c*-statistic is similar to the area under the ROC curve (Harrell *et al*, 1984). The *c*-statistic for survival data indicates the probability that for a randomly chosen pair of patients, the one having the higher predicted survival is the one who survives longer (Harrell *et al*, 1984). Overall performance of the three and five group classifications was measured by model *χ*^2^. When a model is developed and evaluated on the same data, the performance of the model is usually too optimistic. The optimism can be quantified with statistical methods, known as internal validation techniques (Steyerberg *et al*, 2001). To estimate and correct for the optimism in discriminative ability, the steps taken in the Cox regression and recursive partitioning were internally validated by taking random bootstrap samples (100) (Efron and Tibshirani, 1993; Harrell *et al*, 1996).

## RESULTS

The median follow-up time of surviving patients was 50 months. Disease progression occurred in 680 patients, and 533 patients died. The 5-year PFS was 78% (95% CI 76–79%) and the 5-year overall survival 82% (95% CI 81–84%). Most patients had as primary site testis or retroperitoneum (97%), no NPVM (92%), and good AFP, HCG and LDH levels (84, 87 and 67%, respectively) (Table 2

**Table 2:** Characteristics of 3048 NSGCT patients on the IGCC risk factors

**IGCC risk factors**	**Number of patients (%)**	**5-year survival (%)**	**95% CI (%)**	**HR**	**95% CI**
*Primary site*					
Testis/retroperitoneal	2947 (97)	84	82–85	1	—
Mediastinum	101 (3)	37	27–47	6.1	4.7–7.9

*NPVM*					
No	2808 (92)	85	84–86	1	—
Yes	240 (8)	49	42–55	4.6	3.8–5.6
					
*AFP*					
Good	2559 (84)	85	84–87	1	—
Intermediate	349 (12)	71	66–76	2.1	1.7–2.6
Poor	140 (5)	56	47–65	3.6	2.7–4.7

*HCG*					
Good	2656 (87)	86	84–87	1	—
Intermediate	238 (8)	65	58–71	3.0	2.3–3.8
Poor	154 (5)	48	39–56	5.0	3.9–6.4

*LDH*					
Good	2036 (67)	89	88–91	1	—
Intermediate	977 (32)	68	65–71	3.3	2.8–3.9
Poor	35 (1)	51	34–67	6.2	3.9–10.1

Total number subjects	3048 (100)	82	81–84	—	—

NPVM=nonpulmonary visceral metastases.

). All risk factors were predictors of survival as indicated by the Hazard ratios ranging from 2.1 to 6.2, where the tumour marker AFP was the weakest risk factor in the univariable analysis.

### Alternative classifications

The regression-based weights of the risk factors in classification 5R, and the cutoff points on the resulting sum score are presented in Table 3

**Table 3:** Weights, coding of variables, and cutoff on the max function of the IGCC classification and the sum score of the regression-based classification 5R

**Classification**		**IGCC**	**5R**
**Risk factors in the model**	**Coding of risk factors**	**Implicit weights**	**Regression weights**
Primary site	Testis/retroperitoneal	0	0
	Mediastinum	2	15

NPVM	No	0	0
	Yes	2	7

AFP	Good	0	0
	Intermediate	1	2
	Poor	2	3

HCG	Good	0	0
	Intermediate	1	9
	Poor	2	11

LDH	Good	0	0
	Intermediate	1	7
	Poor	2	9

Cutoff points	Good	Max 0	Sum 0
	Intermediate	1	2–10
	Poor	⩾2	⩾11

NPVM=nonpulmonary visceral metastases.

, with the weights and cutoff points of the IGCC classification.

The weights suggest that differences between risk factors were present. Tumour marker AFP had a much lower weight in the multivariate analysis than tumour markers HCG and LDH. As a result, a poor AFP level (score 3) is not sufficient to be classified as poor prognosis in classification 5R. Also, the combination of two or three intermediate tumour markers, which would lead to an intermediate prognosis in the IGCC classification, results in a score of over 10 and thus in classification in the poor prognosis group in classification 5R. The presence of risk factor NPVM (score 7) alone was not sufficient to be classified as poor prognosis, in contrast with the IGCC classification. Patients would only be classified as poor prognosis when other risk factors besides NPVM or AFP are present.

**Figure 1:**
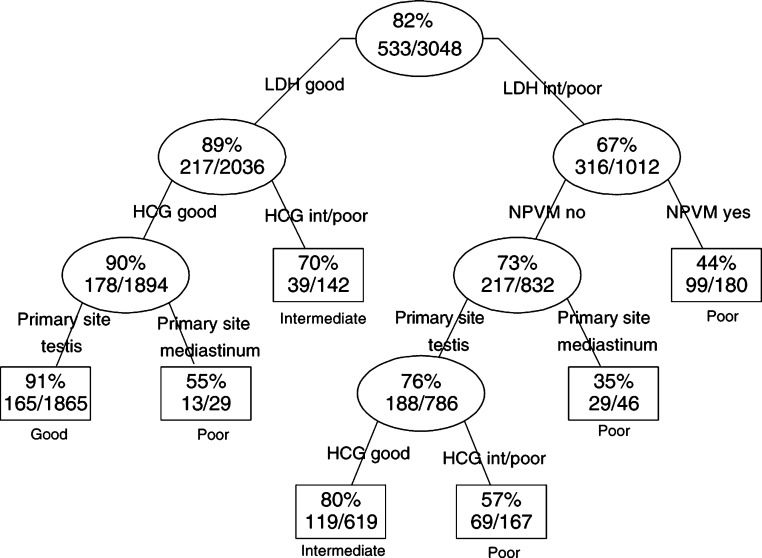
The final tree fitted by recursive partitioning, using the exponential scaling method. The 5-year survival rates, events and total number of observations per subgroup are given. The resulting subgroups are displayed in rectangulars and determine classification 5T.

We identified four significant interactions in the Cox regression model; between AFP and primary site (*P*<0.001), AFP and NPVM (*P*<0.01), HCG and NPVM (*P*<0.003) and HCG and LDH (*P*<0.01). The regression coefficients all had negative signs, indicating that the effect of the risk factors together was smaller than the sum of their separate effects. For all 108 combinations of the IGCC risk factors, we present 5-year survival estimates from the Cox regression model with interactions (Appendix). Patients with testis as primary site and good or intermediate tumour markers had the highest estimated survival (55–92%). Patients with mediastinum as primary site and NPVM had the worst estimated survival (0–64%). Since the number of patients with more than one poor risk factor was limited, the survival estimates for these patients were less reliable. Recursive partitioning resulted in a tree with seven subgroups with 5-year survival ranging from 35 to 91% (Figure 1

), forming the basis of classification 5T. Tumour marker LDH was the principal determinant of 5-year survival, making a split between good LDH (*N*=2036) and intermediate/poor LDH (*N*=1012). The majority of the ‘good LDH’ subgroup consists of patients with no risk factors (*N*=1865) with an observed 5-year survival of 91% (95% CI 90–93%). Furthermore, a subgroup of 29 patients with primary site mediastinum had a 5-year survival of 55% (95% CI 34–72%) and patients with intermediate or poor HCG (*N*=142) had a 5-year survival of 70% (95% CI 61–77%). Within the subgroup intermediate/poor LDH, four further subgroups were identified with the risk factors NPVM, primary site and HCG, with 5-year survival ranging from 35 to 80%.

### Performance

The 5-year survival rates for the good, intermediate and poor prognosis groups were comparable for the IGCC classification and classifications 5R, 5Ri and 5T (Table 4

**Table 4:** Survival of the IGCC classification, the regression-based classifications 5R and 5Ri and classification 5T based on recursive partitioning

	**IGCC**		**5R**		**5Ri**		**5T**	
**Group**	**Surv (%)**	***N***	**Surv (%)**	***N***	**Surv (%)**	***N***	**Surv (%)**	***N***
Good	92	1691	92	1691	92	1691	91	1865
Intermediate	81	862	80	872	80	915	78	761
Poor	50	495	50	485	47	442	49	422

Surv=5-year survival.

). The *c*-statistic of the IGCC classification was 0.732. The apparent *c*-statistics of classifications 5R, 5Ri and 5T were 0.732, 0.735 and 0.718, respectively. Validation showed minor optimism in the *c*-statistic in the Cox regression models (0.002). More optimism was present in the classification 5T, with the *c*-statistic decreasing from 0.718 to 0.709. Classification 5R did not show an improvement in model *χ*^2^ compared to the IGCC classification (model *χ*^2^ 402 and 401, respectively, 2 d.f.). Classifications 5Ri did show a statistically significant increase in overall performance over the IGCC classification (model *χ*^2^ 422, 2 d.f.). Classification 5T had a worse overall performance with a model *χ*^2^ of 374 (2 d.f.).

### Identification of more subgroups

Within the max score, different weights did not lead to an improvement in overall performance over the weights of the IGCC classification (model *χ*^2^ 402, 2 d.f.). The following weights were allocated to derive a max function with five prognostic groups in the IGCC classification with the score varying between 0 and 4; primary site mediastinum (4), NPVM (3), AFP good/intermediate/poor (0/1/2), HCG good/intermediate/poor (0/2/3) and LDH good/intermediate/poor (0/1/3). The 5-year survival varied from 37 to 92% for the five groups of the IGCC classification, from 34 to 92% for classification 5R, from 36 to 92% for classification 5Ri and from 35 to 91% for classification 5T (Table 5

**Table 5:** Survival of subgroups within the IGCC classification, the regression-based classifications 5R and 5Ri and classification 5T based on recursive partitioning

	**IGCC**		**5R**		**5Ri**		**5T**	
**Group (Surv)**	**Surv (%)**	***N***	**Surv (%)**	***N***	**Surv (%)**	***N***	**Surv (%)**	***N***
Good (⩾90%)	92	1691	92	1691	92	1691	91	1865
Intermediate (75–89%)	82	684	81	824	82	818	80	619
Good–poor (60–74%)	72	251	65	225	63	194	70	142
Intermediate–poor (40–59%)	51	321	48	169	51	188	51	376
Poor–poor (⩽40%)	37	101	34	139	36	157	35	46

Surv=5-year survival.

**Figure 2:**
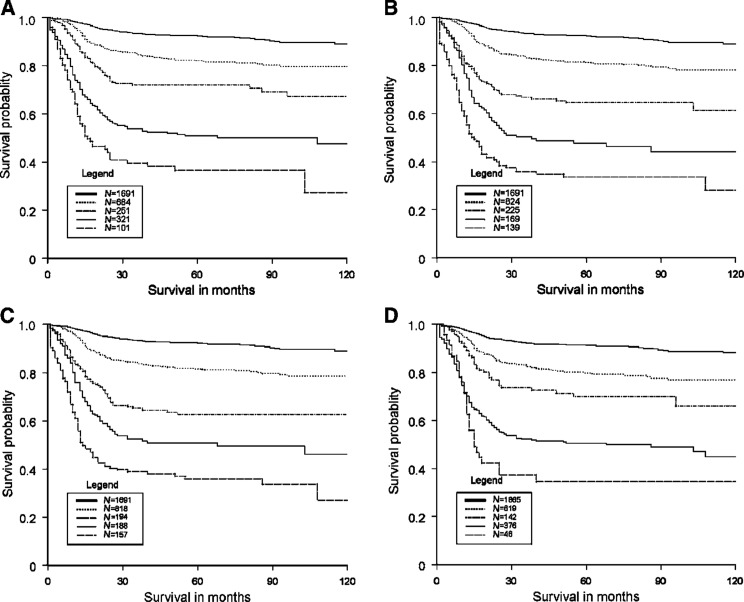
Survival curves for the five groups of the IGCC classification (**A**) and classifications 5R (**B**), 5Ri, (**C**) and 5T (**D**).

Cutoff points on sum score classification 5R: Good 0, Intermediate 2–9, Good–poor 10–16, Intermediate–poor 17–22, Poor–poor >22.

). The cutoff points on the sum score for the five groups of classification 5R are also given in Table 5. The difference in survival between the prognostic groups for each classification is illustrated in Figure 2

. The *c*-statistic for the five groups of the IGCC classification and classifications 5R and 5Ri was slightly higher than for the three group classifications (0.739, 0.741 and 0.744, respectively) and with a small amount of optimism (0.002) for the Cox regression models. The increase of the *c*-statistic for the five groups of classification 5T was very limited (0.722) with an optimism of 0.011. The increase in model *χ*^2^ was more substantial; 422 for the extended IGCC classification, 446 for classification 5R, 450 for classification 5Ri. The increase in model *χ*^2^ for classification 5T (383) was less substantial.

## DISCUSSION

The discriminative ability of classifications derived through Cox regression and recursive partitioning was in concordance with the IGCC classification and therefore supports the validity of the IGCC classification. We did, however, find that not all intermediate tumour markers and poor risk factors were equally important, and that taking these differences into account does affect the classification of patients. In Cox regression-based classifications, especially risk factors NPVM and AFP had less impact compared to the other risk factors. That AFP is of less importance than the other risk factors is confirmed by recursive partitioning where AFP was not selected in the final tree. Furthermore, not all risk factors had statistical interactions. In classifications 5Ri and 5T, only a limited number of interactions were included. Combining several risk factors led to differences in 5-year survival, that is, patients with one poor risk factor had a better chance of survival than patients with three risk factors. These deviations from the weights used by the IGCC classification did, however, not lead to improvements in discriminative ability, in contrast with what we expected. The use of Cox regression and recursive partitioning did allow for more flexible classifications with more subgroups, leading to a small improvement in discriminative ability and 5-year survival of 34% for the poorest risk patients.

It appears that the maximum discriminative ability might have been reached with the current IGCC risk factors and coding, making further improvement in discriminative ability difficult. The risk factors selected for the IGCC classification are in agreement with risk factors used in other studies on identifying good and poor prognosis patients with NSGCT (Bajorin *et al*, 1991; Mead *et al*, 1992). Some other potentially useful risk factors include age, lung metastases and abdominal mass size. However, adding these three risk factors to the Cox model had no substantial effect on discriminative ability (*c* increased from 0.73 to 0.74). One could also consider using continuous codings of tumour markers, but this would lead to an undesirable increase in complexity and decrease in applicability.

The division into more prognostic groups is similar to another division by recursive partitioning of poor prognosis patients (Kollmannsberger *et al*, 2000). Kollmannsberger *et al* identified three prognosis groups: a good-poor, intermediate-poor and poor-poor risk group with 2-year survival rates of 84, 64 and 49%, respectively. These survival rates are higher than the survival rates of the good-poor, intermediate-poor and poor-poor risk groups identified in the IGCC dataset. This may be due to the difference in survival for the poor prognosis patients (72 *vs* 50%), and remains when the difference in follow-up time is taken into account (2 *vs* 5 years). The data in Kollmannsberger *et al* (2000) are more recent and improvements in treatment may have led to the difference in survival.

The lack of improvement in discriminative ability in both the classifications with three and five groups might also be explained by the dominance of the good prognosis group, which has a similar survival for all classifications and contains more than half of all patients. We therefore examined whether discriminative ability increased within the poor prognosis group of each classification. Discriminative ability increased from 0.50 to 0.60, 0.63, 0.64 and 0.65 for the three poor prognosis groups of classifications 5T, IGCC, 5R and 5Ri, respectively. Hence, some improvement was noted within the IGCC poor prognosis group. Furthermore, even though the *c*-statistic is often used and easy to interpret, it is not suitable for detecting small differences in discriminative ability (Harrell *et al*, 1996; Steyerberg *et al*, 2000).

Although the use of Cox regression and recursive partitioning did not have a major effect on discriminative ability, they can still be useful tools in the construction of future prognostic classifications when other criteria are taken into account. One of the advantages of classifications such as the IGCC classification is its simplicity. Classification 5T is reasonably simple with only a few subgroups and the survival probability readily available. Classification 5R is slightly more complicated because of the sum score that has to be calculated. Finally, classification 5Ri is not so much complicated as visually unattractive. Furthermore, survival estimates for infrequent combinations of risk factors are not reliable and therefore provide little information on the prognosis of patients with these risk factors.

A disadvantage of the IGCC classification is its inflexibility. More groups could be defined, but not in a straightforward manner. Classification 5R and classification 5Ri are very flexible with many possible cutoff points. Classification 5T is less flexible due to the limited number of subgroups, but flexibility could be increased by putting fewer restrictions on the recursive partitioning allowing for more subgroups to be identified.

The IGCC classification considered not just discrimination but also simplicity and the size of the resulting prognostic groups and was chosen by consensus from a shortlist of possible models, which balanced these considerations. Consequently, in the IGCC classification there is a lack of transparency; it is unclear how the classification was constructed statistically because statistical considerations were not the only criteria used to derive the classification. Classification 5T shows very clearly how the subgroups were derived from the successive splits in the risk factors. Classification 5R shows the difference in importance between the risk factors and how the risk factors are combined in a sum score. Classification 5Ri could be presented in a similar way as classification 5R, but interpretation of the main and interaction effects is difficult.

The IGCC dataset suffers from a number of limitations. First, not all data were used for the multivariable regression models because of missing data. When patients with missing data differ from the other patients on prognosis, this causes a bias in the regression coefficients and the estimated 5-year survival rates (Little, 1992; van Buuren *et al*, 1999; Clark and Altman, 2003). Secondly, we could not internally validate the IGCC classification, because the exact steps taken in the modelling process (selection and categorisation of risk factors) were not defined. The IGCC classification was applied to a 30% validation set (IGCCCG, 1997), and although the proportion of patients in each prognostic group was similar, the 5-year survival for poor prognosis patients was higher (57%). We did internally validate the modelling steps of the Cox regression models and found minor optimism in discriminative ability. Classification 5T, based on recursive partitioning, however, showed optimism in discriminative ability, as might be expected from a more data-driven method. This, in combination with the poorer performance, suggests that recursive partitioning is less suitable for the construction of prognostic classifications. It can be useful, however, for exploratory analyses in finding interactions between risk factors.

The survival estimates of the IGCC classification were also externally validated with more recent data from an MRC/EORTC trial (*N*=300). The 2-year PFS outcome largely corresponded with the IGCC estimates (IGCCCG, 1997). To gain further insight in the generalisability of the Cox regression models as well as the IGCC classification, further external validation is necessary, in larger recent datasets with longer follow-up.

In conclusion, the IGCC classification appears to be a valid way to classify patients with NSGCT in three prognostic groups. Recursive partitioning is less suitable for the construction of prognostic classifications, because of its poorer performance. Although Cox regression did not lead to a clear improvement in performance, it gave a more flexible and transparent scoring system without much loss in simplicity. We therefore recommend the use of regression-based weights in the development of future prognostic classifications.

## Appendix

Table 6

**Table 6:** 5-year survival estimates and number of patients are given for all 108 combinations of the IGCC risk factors based on a Cox regression model of the IGCC risk factors and interactions AFP and primary site, AFP and NPVM, HCG and NPVM, and HCG and LDH

			**Primary site**				**Primary site**			
			**Testis**				**Mediastinum**			
			**NPVM**		**NPVM**		**NPVM**		**NPVM**	
			**No**		**Yes**		**No**		**Yes**	
**AFP**	**HCG**	**LDH**	**Surv (%)**	***N***	**Surv (%)**	***N***	**Surv (%)**	***N***	**Surv (%)**	***N***
		Good	92	1691	79	27	53	14	18	1
	Good	Intermediate	83	459	60	31	25	12	2	10
		Poor	73	11	43	3	10	0	0	1
		Good	77	81	54	9	15	3	1	0
Good	Intermediate	Intermediate	66	62	38	16	5	1	0	1
		Poor	60	2	30	0	2	0	0	0
		Good	64	16	39	8	4	0	0	0
	Poor	Intermediate	59	56	32	38	2	1	0	2
		Poor	61	0	35	3	3	0	0	0

		Good	88	121	79	5	65	8	44	1
	Good	Intermediate	76	104	60	18	39	14	17	6
		Poor	64	0	43	1	21	0	5	0
		Good	69	16	54	1	28	0	12	0
Intermediate	Intermediate	Intermediate	55	19	37	9	13	0	3	0
		Poor	48	1	30	3	8	0	2	0
		Good	52	2	38	1	11	0	4	0
	Poor	Intermediate	46	13	32	3	7	0	2	0
		Poor	49	3	35	0	9	0	3	0
										
		Good	81	16	76	5	71	4	64	1
	Good	Intermediate	63	43	55	24	48	17	38	3
		Poor	47	2	37	3	30	0	20	0
		Good	54	4	49	0	37	0	32	0

Poor	Intermediate	Intermediate	37	10	31	0	20	0	16	0
		Poor	29	0	21	0	14	0	10	0
		Good	33	0	33	1	17	0	17	0
	Poor	Intermediate	27	1	26	3	12	1	12	0
		Poor	30	0	29	2	15	0	14	0

Surv=5-year survival; *N*=number of patients.

Classification into three groups; good prognosis 5-year survival >90%, intermediate prognosis 5-year survival 65–89%, poor prognosis 5-year survival <65%.

Classification into five groups; good prognosis 5-year survival >90%, intermediate prognosis 5-year survival 75–89%, good-poor prognosis 5-year survival 60–74%, intermediate-poor prognosis 5-year survival 40–59%, Poor-poor prognosis 5-year survival <40%.
